# A holistic approach to the mycetoma management

**DOI:** 10.1371/journal.pntd.0006391

**Published:** 2018-05-10

**Authors:** Sahar Mubarak Bakhiet, Ahmed Hassan Fahal, Ahmed Mudawi Musa, El Samani Wadaa Mohamed, Rowa Fathelrahman Omer, Eiman Siddig Ahmed, Mustafa El Nour, El Rayah Mohamed Mustafa, Manar El Sheikh A. Rahman, Suliman Hussein Suliman, Mohamed A. Gadir El Mamoun, Hajo Mohamed El Amin

**Affiliations:** 1 The Mycetoma Research Centre, University of Khartoum, Khartoum, Sudan; 2 Department of Molecular Biology, Institute of Endemic Diseases, University of Khartoum, Khartoum, Sudan; 3 Department of Medical Imaging, Soba University Hospital, University of Khartoum, Khartoum, Sudan; 4 Department of Biostatistics, Faculty of Mathematical Sciences, University of Khartoum, Khartoum, Sudan; 5 Department of Surgery, Faculty of Medicine, University of Khartoum, Khartoum, Sudan; 6 The Minister of Health, Ministry of Health, Sennar State, Sennar, Sudan; 7 Department of Surgery, Faculty of Medicine, Sennar University, Sennar, Sudan; Hospital Infantil de Mexico Federico Gomez, UNITED STATES

## Abstract

Mycetoma, one of the badly neglected tropical diseases, it is a localised chronic granulomatous inflammatory disease characterised by painless subcutaneous mass and formation of multiple sinuses that produce purulent discharge and grains. If untreated early and appropriately, it usually spread to affect the deep structures and bone resulting in massive damage, deformities and disabilities. It can also spread via the lymphatics and blood leading to distant secondary satellites associated with high morbidity and mortality. To date and despite progress in mycetoma research, a huge knowledge gap remains in mycetoma pathogenesis and epidemiology resulting in the lack of objective and effective control programmes. Currently, the available disease control method is early case detection and proper management. However, the majority of patients present late with immense disease and for many of them, heroic substantial deforming surgical excisions or amputation are the only prevailing treatment options. In this communication, the Mycetoma Research Center (MRC), Sudan shares its experience in implementing a new holistic approach to manage mycetoma patients locally at the village level. The MRC in collaboration with Sennar State Ministry of Health, Sudan had established a region mycetoma centre in one of the endemic mycetoma villages in the state. The patients were treated locally in that centre, the local medical and health personals were trained on early case detection and management, the local community was trained on mycetoma advocacy, and environmental conditions improvement. This comprehensive approach had also addressed the patients’ socioeconomic constraints that hinder early presentation and treatment. This approach has also included the active local health authorities, community and civil society participation and contributions to deliver the best management. This holistic approach for mycetoma patients’ management proved to be effective for early case detection and management, optimal treatment and treatment outcome and favourable disease prognosis. During the study period, the number of patients with massive lesions and the amputation rate had dropped and that had reduced the disease medical and socioeconomic burdens on patients and families.

## Author summary

Mycetoma enjoys all the neglected tropical diseases (NTDs) characteristics. It frequently affects the poorest of the poor in poor communities in remote regions. The affected population are of low socio-economic status, of low visibility and low political and social voice and hence they are neglected. The disease is considered as a social stigma in particularly among females and children thus they tend to hide it for prolong period and when they are compelled to seek medical care the condition is then at a late stage. The mycetoma patients have many financial constraints that hinder them from seeking medical and health care. In the remote mycetoma endemic areas, the health and medical facilities are meagre, and it is difficult for the patients to reach the regional health centres and thus the majority of patients present with late advanced disease. To overcome these treatment difficulties, the MRC had adopted this holistic management approach to decentralised the patient's care, improve the disease awareness and advocacy, provide free medical and surgical treatment locally at the village level, and to improve the affected villages environmental and hygienic conditions. In this communication, the MRC is reporting on this unique experience, discussing the advantages and difficulties faced it and suggesting recommendations to improve it to be adopted worldwide. Reviewing the medical literature revealed, no report on such management approached for mycetoma patients and thus it worth reporting it.

## Introduction

Mycetoma is a common neglected tropical disease, reported worldwide but endemic in many tropical and subtropical regions in what is known as the mycetoma belt and Sudan seems to have the highest endemicity [1, 2]. It is a chronic granulomatous inflammatory disease caused by several true fungi or certain actinomycetes, and hence it is classified as eumycetoma and actinomycetoma respectively [3, 4]. More than 70 organisms are incriminated in causing mycetoma [5, 6]. It is believed that these causative organisms, which are soil inhabitants, are implanted in the subcutaneous tissue via traumatic inoculation [7, 8]. Mycetoma usually spread to involve the skin, deep structures and bones leading to devastating destruction, deformities and disability [9, 10]. Early localised disease is amenable to cure and good prognosis, however, the late advanced disease is characterised by high morbidity and can be fatal [11, 12, 13]. Mycetoma has serious medical, health and socioeconomic bearings on patients, families, and communities particularly in endemic areas [14, 15, 16].

To date, its global incidence and prevalence are not well documented as mycetoma is a neglected disease, not a notified or a reportable one. Furthermore, in most of the endemic regions, there is no proper disease surveillance system, especially in Sudan. Thus most of the reported cases are limited to anecdotal case reports and passive case detection [17, 18]. Moreover, the disease susceptibility, resistance and route of infection are not well characterised [19, 20]. Nevertheless, mycetoma is commonly seen in communities of poor hygiene and environmental conditions where population live in proximity to animals and their dungs. It is believed that thorn pricks and minor injuries are important routes of mycetoma infection. This is supported by the facts that mycetoma is seen more frequently in the feet of patients of low socioeconomic status, with poor hygiene and in villages with animals enclosures made of thorny trees [21, 22].

Clinically, mycetoma starts as a small painless subcutaneous mass that gradually increases in size, then multiple sinuses with seropurulent discharge that contained grains of different colour and sizes develop [23, 24, 25, 26]. Most patients present late with advanced disease and serious complications due to the painless nature of the disease, patients’ low health education level and lack of health facilities in endemic areas [27, 28, 29]. It affects all age groups, but children and young adults of low socio-economic status are affected most, leading to serious economic and social consequences [30, 31, 32].

The proper treatment of mycetoma depends on mycetoma type and disease extent. Numerous mycological and molecular tests are required to identify the causative organisms, and that include grain microscopy and culture, cyto-histopathological examinations and PCR identification [33, 34, 35, 36, 37, 38]. Various imaging techniques such as X-ray, ultrasound, MRI, CT scans are required to determine the disease spread along the various body planes [39, 40, 41, 42]. However, most of these tests and techniques are invasive, of low specificity and sensitivity, expensive for patients and health providers in endemic areas [37]. Currently, there is no point of care diagnostic test for mycetoma. Patients need to travel for long distances to regional centres to establish the diagnosis, and that is not always feasible due to their low socio-economic status, low health education, and roadblocks in particularly during the raining season.

Patients need prolonged periods of management involving diagnosis, treatment both medical and surgical and regular follow up. Treatment may last at least one year for the minor lesions to resolve and several years for large lesions. Even after full recovery, patients need to be followed up closely for evidence of recurrence, which is not uncommon [43, 44, 45, 46].

The currently available treatments for mycetoma are suboptimal and disappointing, characterised by low cure rate (28%) and high patients’ follow up dropout (54%) rate [47, 48]. In general, actinomycetoma is treated by a combination of antibiotics, and for eumycetoma, a combination of antifungals and wide local surgical excision is needed. The available medication is not very effective, expensive, with many side effects and hence the high patients’ dropout rate [47, 48].

The diagnostic tests and treatment are expensive that amount up to $2500 per year. Whereas to the annual income in Sudan is less than $400 per capita (according to the UNDP, 2006), that creates an enormous economic burden on the patients, their families, community and eventually the whole health system in the country [49].

It is interesting to note, worldwide, there are neither preventive or control measures nor programs for mycetoma [20]. The disease surveillance, especially in Sudan, is limited to targeted prevalence studies, case reports, and passive case detection. The absence of a standardised, centralised mycetoma surveillance system has far-reaching effects on how the existing interventions are delivered in a cost-effective and evidence-based manner.

In summary, mycetoma is a very devastating endemic disease of the most underprivileged population whether socially, economically or in terms of development. Mycetoma patients tend to travel from distant remote parts of the country to central centres for the treatment. This causes high financial burden and delay in treatment initiation. Furthermore, the disabling nature of the disease hinders access to healthcare service for the majority of the patients. Thus health services decentralisation will improve the accessibility and equity of health services to patients and will directly drop the huge financial burden.

With this background, this community-based study was conducted with the objective of applying a new holistic approach to the management of mycetoma patients at the village level. That included setting up a regional mycetoma centre with a telecommunication network, offering free of charge both medical and surgical treatment at the centre, training of medical and health staff on early case detection and management, community health education, improvement villages’ hygiene all these were based on available health system structure and minimum requirement.

## Materials and methods

This is a community-based cross-sectional study which was conducted at Eastern Sennar Governate, Sennar State, Sudan in the period 2015–2017. The Governate is 400 km south of Sudan capital Khartoum. It has 292 villages with a total population of 219,800 inhabitants. In this study, 19 villages in the Governate were surveyed for early mycetoma patients’ detection and management. One village; Wad EL Nimear, the highest endemic village, was studied in depth and compared to another village Wad Onsa which is two kilometres apart with less disease prevalence, Table 1, S1 Map. During the study period, four medical and health mobile missions were organised and conducted by the MRC to the studied villages. The mission team consisted of three consultants surgeons, one physician, two consultants radiologists, four surgical registrars, two radiology registrars, one anaesthesiology registrar, one molecular biologist, one epidemiologist, one environmental consultant, one pharmacist, five medical officers, three surgical scrub nurses, two anaesthetic assistants, four laboratory technologists, two nurses, 10 medical students, one photographer, two fine artists and one musician. The study was implemented in partnership with Federal Ministry of Health, Sennar State Government, the local administrative authority and local community leaders and activists to assure the services sustainability and the study outcome execution.

**Table 1:** The Wad Onsa and Wad EL Nimear villages characteristics.

Characteristics	Wad Onsa village	Wad EL Nimear village
Houses	Houses reasonably organised, spaced, made of mud or bricks	Not organised, overcrowded, made of mud and animals dungs
Animals enclosures	Commonly made of iron, Located outside the houses	Commonly made of thorny trees and bushes, within the houses
Animals dungs	Few, outside the houses	Plenty within the houses
Thorny Trees and bushes	Few in certain parts	Plenty at different parts
Hygiene standard	Reasonable	Poor
Sanitation	Reasonable	Poor
Personal hygiene	Reasonable	Poor

### Early case detection

Early case detection and management required house to house total coverage survey. That was conducted in 19 villages in the Eastern Sennar Governate. The data were collected by well-trained teams of medical officers, house officers, medical students, health care providers and community activists using a digital pre-designed validated closed-ended questionnaire in smart tablets. Computer Assisted Patients Identifier (CAPI) a computer application which was designed for this study was used. To validate the study questionnaire and the CAPI, a small pilot study was conducted before the data collection in a nearby village.

**Fig 1:**
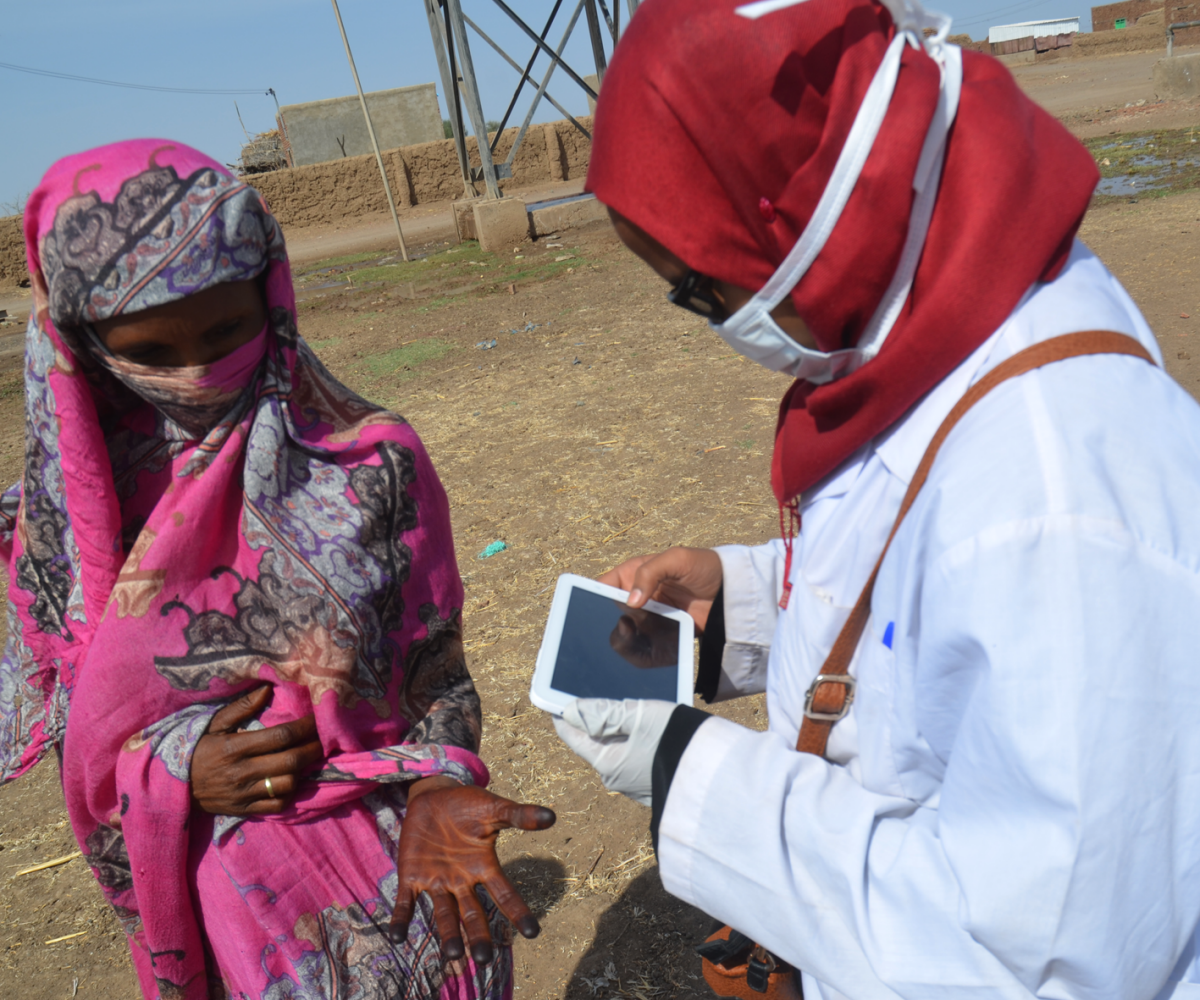
Photograph showing a surveyor using a computer tablet with the CAPI programme to collect information on a suspected patient.

The CAPI is a computer application predesigned for this study to collect data from the study villages and suspected patients. It was designed by an information and technology expert from the Faculty of Mathematical Sciences, University of Khartoum. It was used in computer tablets or smartphones. It can be used offline and online. CAPI was connected to the MRC, Data Centre system and the data analysis was performed spontaneously, the results can be displayed on Google maps, Fig 1.

The data collection questionnaire had included the suspected patient’s demographic characteristics; name, age, state and village localities, lesion site, the presence of mass, sinuses, grain colour, contact address, lesions photographs, the suspected patient’s locality geographic coordinates (latitude, longitude, altitude) and the neighbourhood photographs.

**Fig 2:**
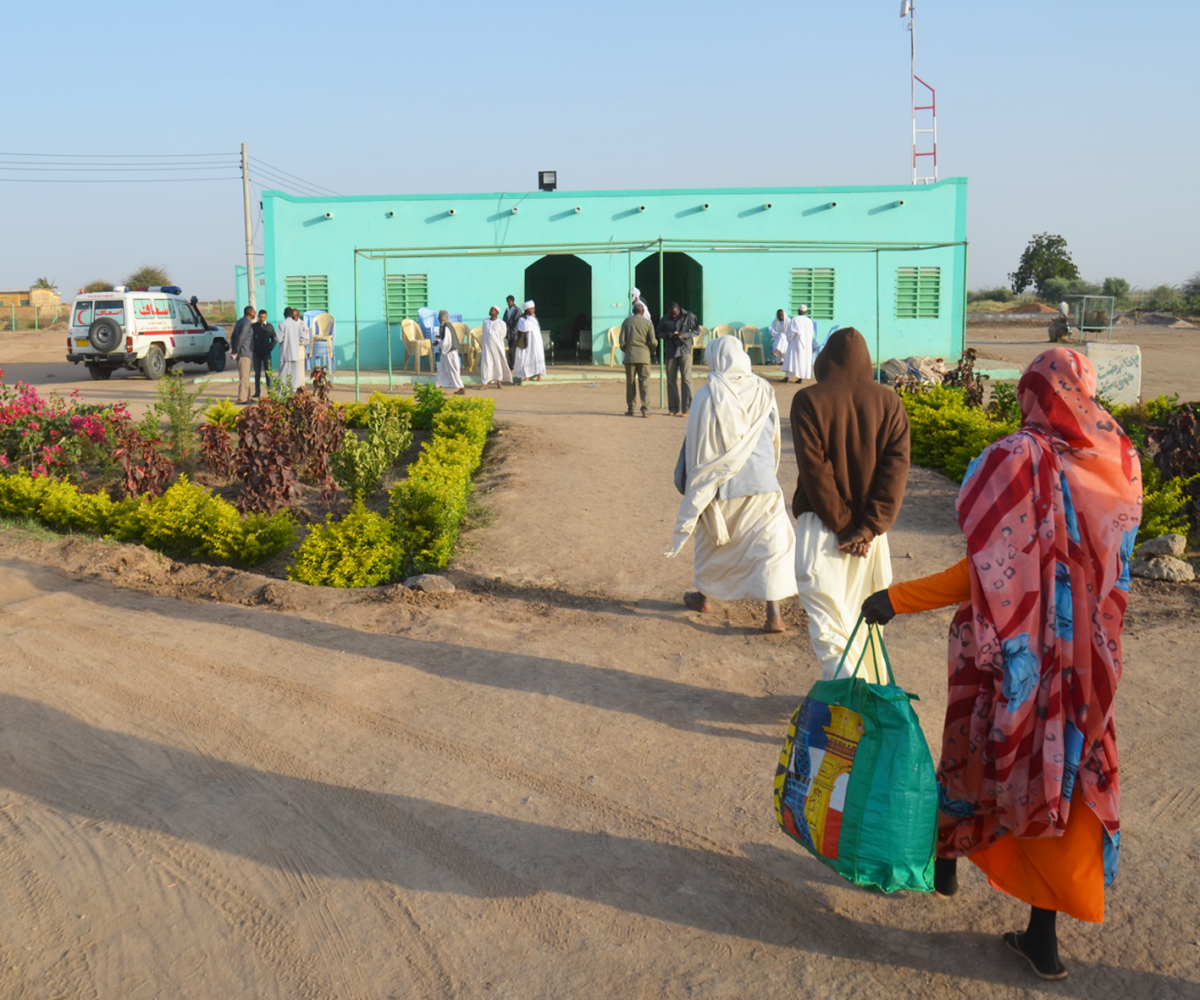
Photograph showing Wad Onsa regional mycetoma centre at East Sennar Governate.

All suspected mycetoma patients from the Governate were referred to Wad Onsa Mycetoma Regional Center (WOMRC). The WOMRC was established in 2015 as a partnership between the Federal Ministry of Health, Ministry of Health, Sennar State, MRC and the local community to manage mycetoma patients locally in their region. The centre consists of small surgical operation complex, two wards, pharmacy, laboratory, ultrasound and out-patient suites and telemedicine facility connecting the WOMRC and the MRC, Fig 2.

At WOMRC centre, the patients were managed by the MRC mobile mission team with continuity of care provided by a surgical team from the regional Sennar Teaching Hospital and the resident doctor at the WOMRC in direct contact with the MRC team via the telemedicine facility.

The diagnosis of mycetoma was established by careful clinical examination and lesion ultrasound examination by mobile ultrasound machine (Paolus–UF-760AG) conducted by the consultant radiologists. All mycetoma suspected patients underwent surgical excisions under general or spinal anesthesia by the consultants surgeons and surgical registrars. The histological examination of the surgical biopsies and grains culture were performed at the MRC in Khartoum as described previously [33, 35]. Some patients with massive lesions were referred to the MRC for further assessment and management. All the investigations and treatment were provided free of charge. The Sennar Ministry of Health had provided free meals and transportation for the patients and their families

The confirmed mycetoma patient’s information was entered into a predesigned patient management record. This included patient’s demographic characteristics, diagnostic tests results, management decisions, treatment received, follow-up and final patient treatment results. This information was regularly checked and updated throughout the patient management and follow-up period.

A system for medicines and consumables procurement, delivery and storage was designed. The medicines included antifungals, antibiotics, analgesics, intravenous fluids and anesthetic medicines as well as the surgical and anesthetic consumables. The medicines were procured from the Central Medical Supply Corporate in Khartoum, shipped and stored at the WOMRC pharmacy at optimum conditions. They were dispensed by the local assistant pharmacist. Patients living in remote areas, of low socio-economic status and unable to attend the outpatient's clinic at WOMRC usually receive their medicines by a community activist who dispensed them use a toktoko (large motorcycle). The patients’ information, the medicines doses and quantities were regularly registered. All these information and procedures were documented and regularly reported to the MRC.

### Health care providers training

**Fig 3:**
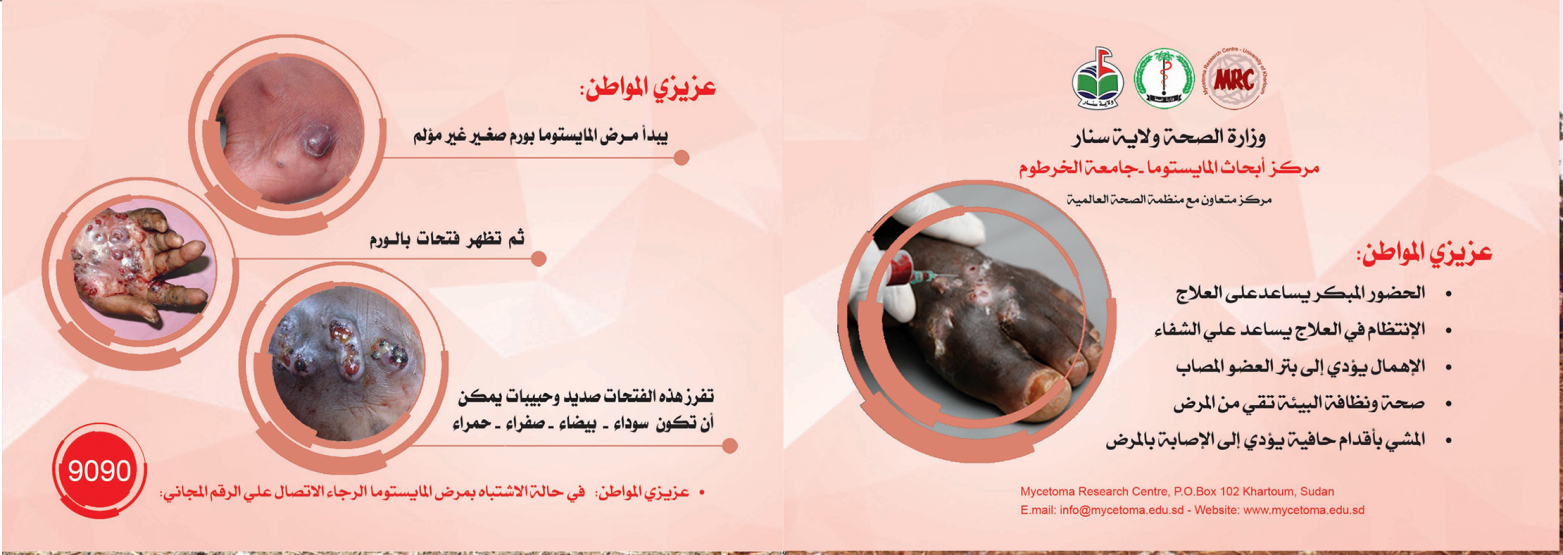
Suspected patients’ referral card.

More than 300 care providers; medical assistants, nurses and public health officers were trained on different aspects of mycetoma, which included the disease causation, presentation, diagnosis and treatment, patients’ care, referral indications and system, community health education and disease advocacy. The instructional training methods included presentations, group discussions, clinical sessions and ultrasound diagnosis demonstration. Suspected patients’ referral card was designed and distributed to the trainee, Fig 3. The improvement in knowledge, attitude and practice (KAP) of the trainees was assessed by pre and post-training tests S1 File. The training sessions were conducted at Singa Town, Sennar State capital, WOMRC and in various East Sennar Governate villages.

Medical students from the local university; Sennar University, as well as the University of Khartoum and other health institutes were training at WOMRC, on early patients’ detection, referral and management and the CAPI use was conducted

To gain the Sennar State political involvement and support, the training sessions were addressed by the Sennar State Governor and the Minister of Health, Eastern Sennar locality Governor and community leaders.

### Local community involvement

**Fig 4:**
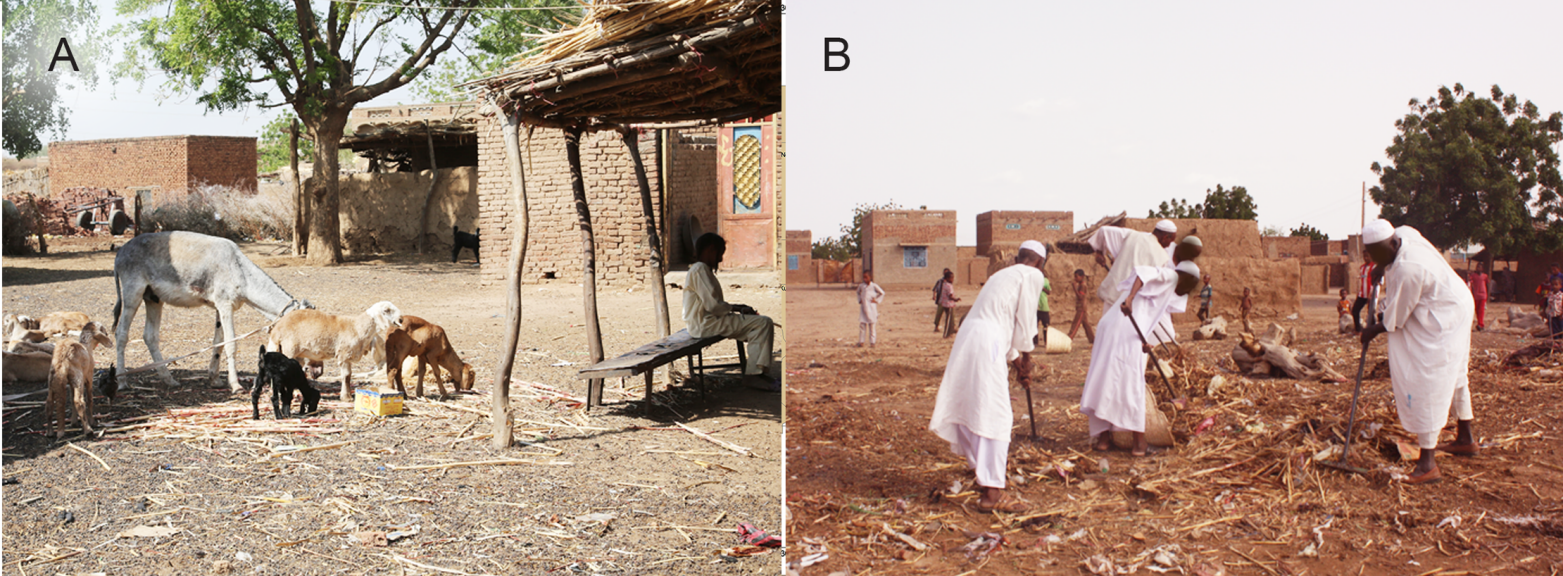
(A) Photograph showing the poor community environment, animals in proximity with the villagers within the houses, animals’ dungs and dirt. (B) The villagers removing the thorny animals’ enclosures, dirt, animals’ dungs and burring them to improve the local environment and hygiene.

Several meetings with the local villages’ leaders, villagers and community activists were conducted at the Wad Onsa village leader’s home, the village’s mosque and WOMRC to explain the study objectives and to gain their confidence and support. They were actively involved in the mycetoma advocacy and awareness activities. The local Red Crescent volunteers were trained in mycetoma advocacy and took an important role in improving the local environment and hygiene in the affected villages, Fig 4.

A toktok was donated by the MRC to a community activist at Wad EL Nimear village for transporting patients and their medicines between the different villages and for mycetoma advocacy. In appreciation of excellent, active and energetic involvement in mycetoma advocacy and awareness, three Mycetoma Ambassadors from the Sennar State were selected.

### Health education & advocacy

**Fig 5:**
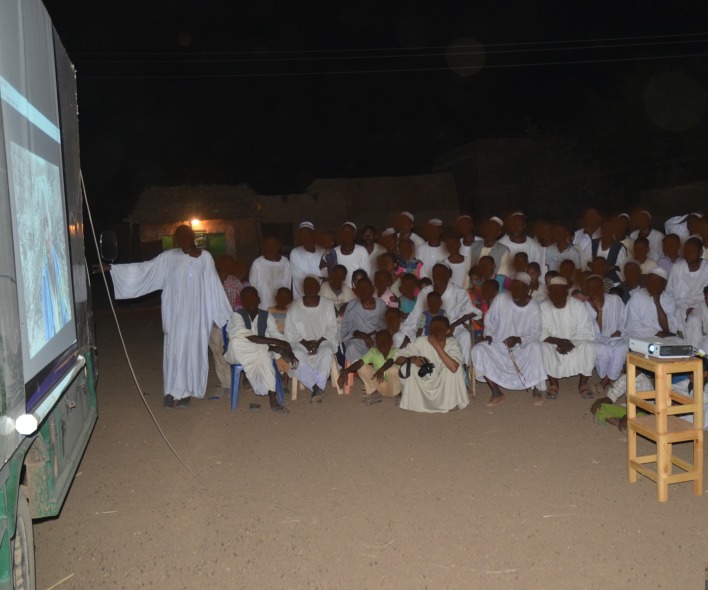
The villagers attending health education session, watching video films on mycetoma awareness.

The study Health Education Team was led by social workers from the Association for Aid and Relief, Japan, Khartoum Office, an active NGO in Sudan with several fine artists, musicians and community volunteers. The health care providers, community leaders and activists, school teachers and medical students from the local university were trained to conduct health education and advocacy sessions. Several health education sessions and activities were carried out for early active case detection were conducted. The sessions included small group discussion, school visits sessions, video films watching, and interactive open theatre drama, Fig 5.

“Mesaket Story”, a drama film documented a mycetoma patient journey from minor infection which was neglected till limb amputation was produced and was shown to more than 2000 individuals at WOMRC and other villages.

### Community environmental improvement campaigns

**Fig 6:**
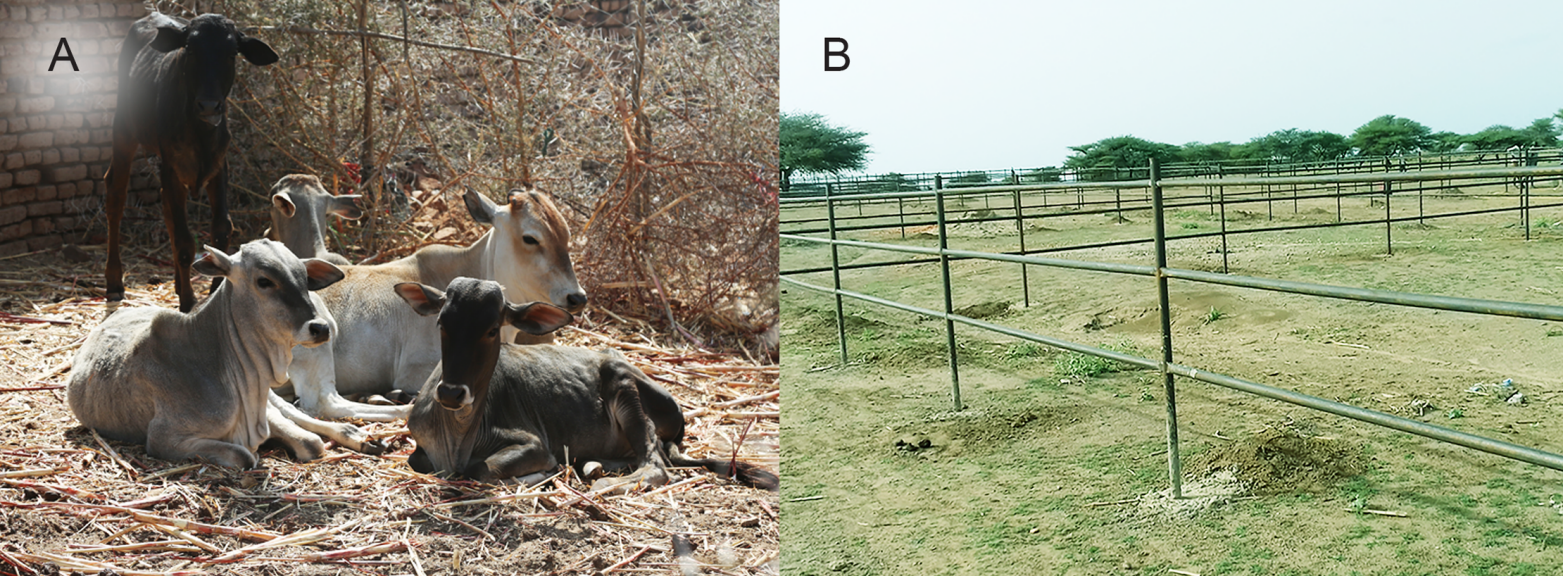
(A) The classical animals made of thorny trees and bushes covered with animals’ dungs, dirt and rubbish. (B)The new modern animals’ cages outside the village.

Several campaigns to improve Wad El Nimear village environment, sanitation, and hygiene to reduce the mycetoma transmission risk factors such as thorns, sharp objects, animals dungs, were organised by the State Government, official local authorities, community leaders and activists, and Red Crescent volunteers in collaboration with the study team. The thorny trees and bushes, thorny animals enclosure, animals dungs, dirt and rubbish, were removed and burnt, Fig 6.

To improve the village hygiene, reduce the contact with the animals and their excreta and to eradicate the thorny cages, 72 modern animal enclosures were constructed outside Wad EL Nimear village. This project was conducted by a kind donation from an engineering company as its social reasonability activity. These new animals cages were distributed free of charge to the villagers, Fig 6.

In mycetoma, the foot is affected most, and traumatic inoculation of the causative organisms which are present in the soil is believed to be the route of infection. The habit of going barefooted in the villages and the minor trauma are considered the risk factors for mycetoma. To reduce these risk factors, the study team has distributed around 800 new shoes to the school pupils at Wad EL Nimear village to improve the personal hygiene and to reduce the risk of developing mycetoma.

### The social impacts of mycetoma

Forty students from the Department of Social Sciences, at the University of Khartoum, spent two weeks at Wad Onsa village studying the social background of the population in the affected villages in the study area and assessed their KAP to mycetoma and its socioeconomic impacts. They surveyed in depth ten villages in the locality. Opened ended questionnaire and focus group discussions were used to obtain the data.

### Project sustainability

A Project Management Board was established headed by the Minister of Health and the senior health officials, Sennar State, the Sennar State Mycetoma control programme officer, local villages’ leaders and activists, local health care providers and MRC representative. The Board oversees the project implementation and update, problem sharing, analysis and solving. The Board has regular meetings to review the quarterly reports to provide advice and recommendations for improvement.

### Ethics statement

The study ethical clearance was obtained from Soba University Hospital Ethical Committee to conduct the study. Informed consents were obtained from the leaders of the villages, informed written consents were obtained from State Ministry of Health and every suspected and confirmed patients. All medical records were anonymised

## Results

### Patients’ management

During the study period, 758 mycetoma suspected patients from the surveyed villages and other villages in Eastern Sennar Governate were seen at WOMRC. All of them had an ultrasound examination of the suspected lesions. Of them, 220 patients had ultrasonic evidence of mycetoma, and they underwent wide local surgical excisions (218 patients), and two patients had amputations. They were 134 males (60.9%) and 86 females (39.1%). Their ages ranged between 2 and 70 years and age group 15–30 years was the most affected one. Most of them were students 68 (30.9%), housewives 46 (20.9%), farmers 35 (15.9%), (Table 2). The geographical distribution was uneven, but Wad El Nimear village had the highest prevalence, (Table 2).

**Table 2:** The patients’ demographic characteristics.

Characteristics	No.	%
**Gander**		
Male	134	60.9
Female	86	39.1
**Age**		
>15 years	51	23.2
15–30 years	106	48.2
31–60 years	61	27.7
>60 years	02	0.9
**Occupation**		
Student	68	30.9
Housewives	54	20.9
Farmers	35	15.9
Workers	05	2.3
Jobless	07	3.2
Other	26	11.8
Missing data	32	14.5
Employee	01	0.5
**Locality**		
Wad El Nimear	40	18.2
Al-Ragal	22	10
Wad Alabas	22	10
Al-Amarat	20	9.1
Wad Onsa	15	6.8
Other villages	101	45.9
**Duration**		
<2 years	63	28.6
2–5 years	96	43.6
6–10	28	12.7
>10	13	5.9
Missing	20	9.1
**Local pain**		
Yes	39	17.7
No	152	69.1
Sometimes	12	5.5
Missing	17	7.7
**Local trauma**		
Yes	38	17.3
No	151	68.6
Not sure	13	5.9
Missing	18	8.2
**Sinuses presence**		
No	142	64.5
Yes	51	23.2
Missing	27	12.3
**Grains discharge**		
Yes	34	15.5
No	161	73.2
Missing	25	11.3
**Site**		
Foot	159	72.2
Hand	59	26.8
Other rare sites	02	01.0
**Size in cm**		
<5 cm	139	63.2
5–10 cm	51	23.2
>10 cm	02	0.9
Missing	28	12.7

Most of the pateints (72.2%) had short disease duration. Pain at the mycetoma site was not a common symptom in these patients; seen in only 39 patients (17.7%). Local trauma at the mycetoma site was reported in only 38 patients (17.3%). Most of the patients had no sinuses (early lesion) 142 (64.5%), and 72 patients (32.7%) had black grains discharge from their sinuses, (Table 2).

The foot 159 (72.2%) and hand 59(26.8%) were affected the most. Less common sites were the back and gluteal one each, (Table 1). The majority of patients 139 (63.2%) had small lesions less than 5 cm in diameter, 51 patients (23.2%) had lesion between 5–10 cm in diameter, and only two patients (0.9%) had lesions more than 10 cm in diameter, (Table 2).

The lesions ultrasound examination findings were mycetoma in 202 patients (91.8%) and foreign body granuloma in 18 patients (8.2%). The surgical procedures performed ranged from wide local excision 218 (99%) to amputation 2 (1%). All patients had an uneventful postoperative recovery. The operatives findings included mycetoma lesions 192 (87.3%), foreign body granulomas with thorns 18 (8.2%), fibroma 2 (1%) and others soft tissue masses. The diagnosis was confirmed by surgical biopsies histopathological examinations, and that showed evidence of eumycetoma in 189 patients (85.9%), foreign body granuloma 17(7.7%), actinomycetoma 3 (1.4%) and others 11(5%). The latter included no-specific granuloma, neuromas and fibromas, (Table 3).

**Table 3:** The patients’ management outcome.

Findings	No.	%
**Ultrasound findings**		
Mycetoma	202	91.8
Foreign Body Granuloma	18	08.2
**Surgical treatment**		
Wide local excision	218	99
Amputation	12	01
**Operative findings**		
Mycetoma	192	87.3
Foreign body granuloma	18	8.2
Fibroma	02	01
Others	08	3.3
**Final diagnosis**		
Eumycetoma	189	85.9
Foreign body granuloma	17	7.7
Actinomycetoma	03	1.4
Others	11	05
**Post-operative Recurrence**		
Yes	37	16.8
No	157	71.4
Miss	26	11.8
Follow up		
Dropout	26	11.8

Most of the patients were followed up at the WOMRC. Thirty-seven patients (16.8%) developed recurrence, due to multifactorial factors which included massive lesion, patients’ non-compliance with treatment or other factors. Twenty-five patients (11.4%) were lost to follow-up.

### Patients’ information system

Confirmed Mycetoma patients’ information was entered into the pre-designed patient’s management records. These records included full details of the patient’s demographic characteristics, diagnostic tests results, the management offered, follow-up and final patient treatment result. This information was regularly monitored and updated throughout the patient journey.

All these data were systematically reported to the MRC in quarterly basis through two types of reporting format; hard copy and a digital one, the latter one was transmitted through a telemedicine facility at the WOMRC and MRC. The reported information was systematically entered in the pre-designed data analysis software for further analysis and systematically checked for information accuracy. Data from management teams, diagnostic services and inventory was crosschecked and discussed regularly to improve recording and reporting process.

### Free medicines management

The current treatment of choice for eumycetoma is itraconazole in a dose of 400mg /day. It costs around 26 US$/day, that is not affordable by neither patients nor local health authorities, and hence the MRC managed to raise funds to procure and dispense itraconazole free of charge to patients at the WOMRC.

A system for medicines procurement, delivery, storage and dispensing at the WOMRC was designed and tested during the study.

### Community mycetoma advocacy activities

A random sample of 218 individuals were tested before and after showing them “Mesaket Story” a drama film. The results showed improvement in their knowledge, attitude and practice and towards mycetoma, (Table 4).

**Table 4:** Mycetoma advocacy KAP study findings before and after film watching.

Questions	Response Before filming	Response After filming
**mycetoma is caused by**		
**Fungus**	51 (23.4%)	19 (7.8%)
**Bacteria**	29 (13.3%)	11 (5.0%)
**Both**	61 (28.0%) **	180 (82.6%) ** *p*
**Viral**	61 (28.0%) **	08 (3.7%)
**Not sure**	16 (7.3%)	00(0%)
**Mycetoma causative organism found in**		
Soil	193 (88.5%) **	214 (98.2%)
Water	15 (6.9%)	03 (1.3%)
Other	00 (00%)	00 (00%)
I do not know	10 (4.6%)	01(0.5%)
**Mycetoma presentation**		
Painless swelling	90 (41.3%)	33 (15.1%)
Sinuses formation	45 (20.6%)	11 (5.0%)
Discharge and grains	61 (28%)	26 (11.9%)
Swelling, sinuses, grains	22 (10.1%)**	148 (67.9%)
**Mycetoma route of entry**		
Through small wound in the skin	65 (29.8%)	13 (6.0%)
Following sharp object or thorns injuries	90 (41.3%)	26 (11.9%)
Poor hygiene, defecation	41 (18.8%)	05 (2.3%)
All of the above	22 (10.1%)	174 (79.8%)** *p*
**Is it infectious**		
yes	96 (44.5%)	31 (14.2%)
No	68 (31.3%)	180 (82.6%)** *p*
Not sure	54 (24.3%)	07 (3.2%)
**Mycetoma is seen in**		
Foot	159 (72.9%)	35 (16.1%)
Hand	28 (12.8%)	02(0.9%)
Back	01 (0.5%)	01(0.5%)
Head	03 (1.4%)	03(1.4%)
Abdomen	07(3.2%)	00(0.0%)
All of the above	20(9.2%)**	177(81.2%)
**Mycetoma treatment**		
Traditional	05 (2.3%)	02(0.9%)
Medical	113 (51.8%)	172 (78.9%)
Both	100(45.8%)	44 (20.2%)
Disease complications		
Difficulty in walking	20(09.2%)	04 (1.8%)
Deep Infections	11(5.1%)	03 (1.4%)
Amputation	137 (62.8%)	26 (11.9%)
All of the above	50 (22.9%)	185 (84.9%)
Prevention measurements		
Shoe wearing during working	209 (95.9%)**	218 (100%)
**Other**	00(00%)	00(00%)
**Not sure**	09(4.1%)	00(00%)

** *p*<0.01

Several small group sessions were organised at different villages, schools, mosques and community clubs. 200 community activists, 50 Red Crescent volunteers and 500 school teachers were trained on mycetoma advocacy and awareness.

The fine artists and musician had organised several interactive open theatre dramas. Different health education materials in different forms were used.

**Fig 7:**
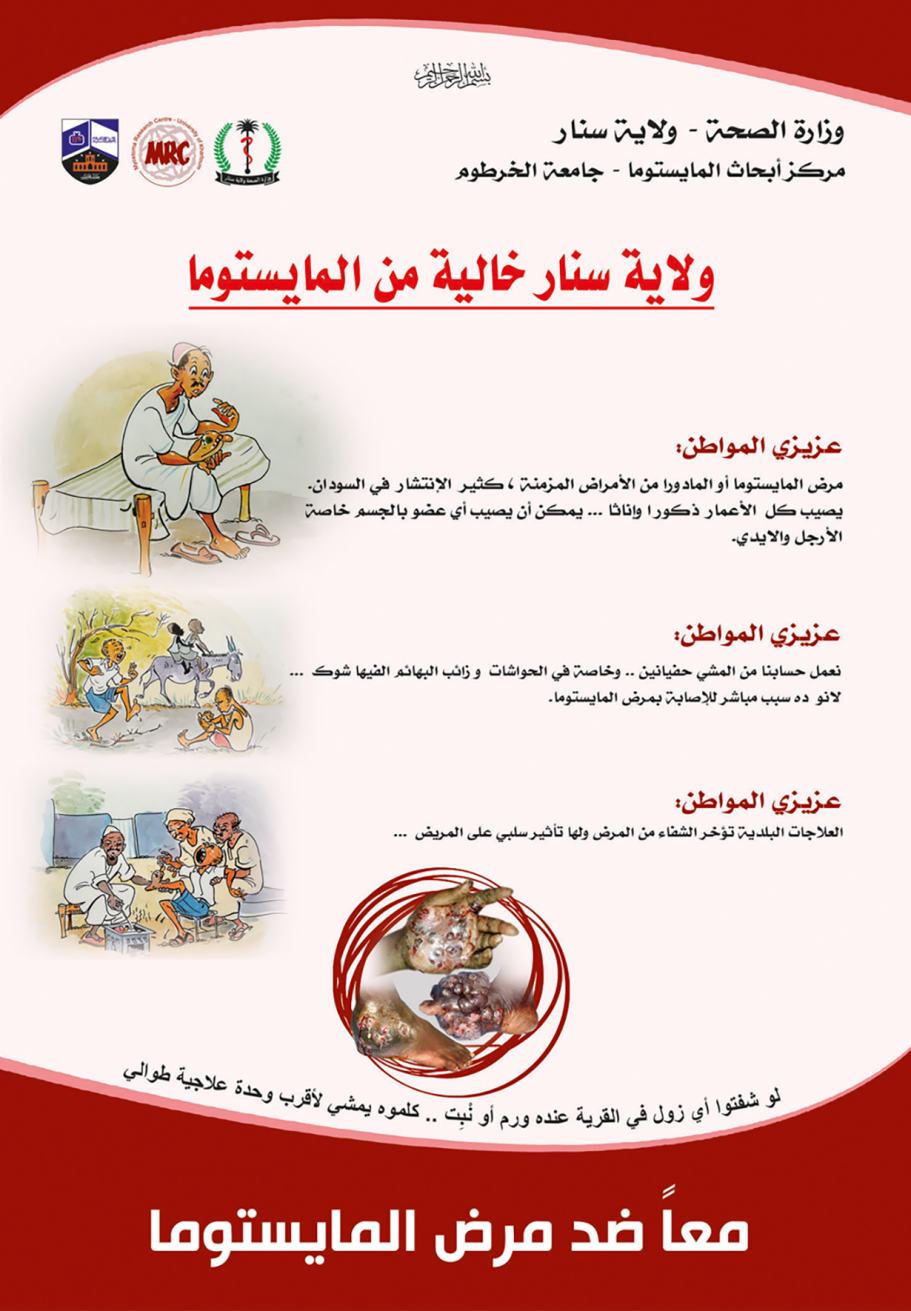
Community leaders, villagers and health and medical staff training material.

Experts in watercolouring, oil painting and photography have greatly contributed to mycetoma advocacy and awareness through their production of high-quality paintings, photographs and videos captured from the studied Governate, Fig 7.

### The social aspects

During the study, six patients with amputations received limb prosthesis donated by the Agent of Aid and Relief, Japan. This had remarkably improved the life quality.

## Discussion

The MRC records showed that the EL Gazeria, White Nile and Sennar States are the top endemic states in the country [28]. Despite been the third, Sennar State has been chosen for the present study due to the strong commitment of the political leaders, civil societies and communities leaders to support and implement the study and its research outcome. The local communities’ leaders were aware of the negative impacts of mycetoma on health and its socioeconomic bearings, and hence their response to the study team requests was swift and extremely positive.

The concept of the village specialised mycetoma centre reported in this communication is a unique one. The WOMRC had delivered integrated medical and social services at the heart of an endemic area. The centre was established as a joint project between the Federal, Sennar State Ministries of Health and the local community, which by itself an exceptional initiative. This study demonstrated numerous positive impacts of the centre on the local communities. It provided local, decentralised mycetoma services in a location with bare minimal health service provision and has improved the local population health education and disease awareness.

The telemedicine which links the MRC in Khartoum with the centre has facilitated the management and follow-up of patients, thus reducing the financial and geographic burdens on the patients and families, and also reduced the patients’ follow-up dropout rate. The dropout reported in this study (11.8%) is less than that reported at the MRC (54%). Although the capital cost of the telemedicine setup is high in developing countries, however, in the long run, it is cost-effective for the patients, families and health authorities in mycetoma endemic regions. This is a unique experience that has not previously been reported for mycetoma or other neglected tropical diseases (NTD) and can be replicated for other endemic NTDs.

The global lack of disease control or elimination programmes due to the unavailability of basic disease epidemiological characteristics has resulted in early case detection and management as the only available method to reduce the disease incidence and prevalence and its community impacts [28]. It is now evident that WOMRC has tremendous bearings on disease management by offering early case detection facilities and free, decentralised medical, health and advocacy services. That is evidenced the fact that many patients (63.2%) with small lesions and patients (72.2%) with disease duration of less than five years were seen at the centre and only two patients underwent amputation during the study period compared patients seen at the MRC [28]. Furthermore, that is supported by the improvement in disease awareness as evidenced by the KAP study results.

The mycetoma onset and progress are usually slow and painless, affecting patients of low socio-economic and health education levels. Hence these patients are different from patients with other deadly infectious diseases, e.g. malaria, cholera, leishmaniasis, where patients have no other choice but to report early and follow medical instructions [50, 51]. Moreover, mycetoma patients with early lesions differ from patients with large disabling mycetoma lesions. Early lesions are usually tiny and painless thus not interfering with their normal daily activities. Some patients consider it at this stage as a trivial or even normal event. These patients usually have many other more pressing social and economic problems than these tiny lesions, e.g. the short busy seasonal farming session, raising children in poor conditions and others. Most of the patients consider these early lesions are not a priority, and in fact, they believe that treatment will delay undertaking other urgent duties, and this explains the late presentation with massive lesions [28,31].

It is evident from this study that our holistic management has addressed many issues. The community engagement activities have led to active early case detection which is supported by the high number of patients with early disease seen at the WOMRC reported in this study. Such patients have a high chance of cure and were amenable to treatment with a good outcome [47,52].

The immediate access to free treatment at the village level has reduced patients’ delay in starting treatment, eliminated patients’ geographical and financial burdens, treatment interruption, and reduced the high follow up dropout rate. Treatment interruption can induce drug resistance. It is therefore vital to ensure sustainability and availability of the free mycetoma treatment services.

The health system in Sudan consists of the three levels; the rural, regional and central levels. The medical assistants, the health care providers, are the backbone for the management of mycetoma patients in Sudan at the rural level. Most of them have poor surgical experience and used to operate on the mycetoma patients under local anesthesia and suboptimal conditions. This practice has led to the high recurrence rate which was documented in many reports [1,2,53]. Recurrent disease is usually associated with wide local disease spread. Hence it is difficult to cure and necessitate repeated surgical excisions, numerous deformities and disabilities [1,2]. At Sennar State, most of the medical assistants were successfully trained on the different aspects of mycetoma care, management and referral.

A well-trained multidisciplinary team on mycetoma care was developed in Eastern Sennar region. It consists of a trained surgeon, surgical theatre attendants, anesthetic assistants, pharmacy assistant, ultrasound technicians, nurses, information technology expert, statistical clerks, community leaders and activists. This is an essential step in providing comprehensive and holistic management for the mycetoma patients.

To date, the definitive route of infection in mycetoma is an enigma. However, it is clear that mycetoma incidence is high in areas of poor environmental conditions, among people with poor personal hygiene and people living in proximity to animals and their dungs and where thorns, dirt and mud prevail. Hence this study aimed to improve the living and hygienic standards of Wad EL Nimear village, one of the highly endemic villages in the locality. The local villagers were encouraged to improve their living conditions. To achieve this goal, many advocacy and awareness campaigns were conducted, and new modern hygienic animals’ cages were constructed and donated to them free of charge. In support of these measures, the local Governate authorities issued a law banning the presence of animals inside the village. The kind donation of the animals’ cages by the engineering company as a response to the intensive mycetoma awareness and advocacy in Sudan.

In this study, the community leaders and activists were actively involved in conveying messages to their community in their own culture and traditions. This was important to accept these holistic disease management procedures. Likewise, the local villagers have actively engaged in promoting their health and improvement of local environmental conditions that believed to be the main source of transmitting mycetoma.

In conclusion, the holistic and comprehensive management approach implemented in this study has improved the mycetoma patients’ quality of care in the studied endemic area. More early disease was detected and treated. The treatment interruption rate was reduced thus increasing the cure rate and decreasing the recurrence and hospitalisation rates. This will eventually lead to decrease in the amputation and disability rates. The results obtained from this study suggest that such a study can be expanded to other endemic areas in the country. The MRC, as a WHO Collaborating centre on Mycetoma, will communicate this experience to the WHO to share it with other mycetoma endemic countries and assist in better management, prevention and control of the disease.

## Supporting information

S1 Checklist STROBE checklist. (DOC)

S1 File The KAP Pre and post-training tests. (PDF)

S1 Map Sudan map showing Sennar state and Wad Onsa and Wad El Nimear villages. (TIF)
