# Supplementary material for: A holistic approach to the mycetoma management
Source: PLoS Negl Trop Dis. 2018 May 10;12(5):e0006391. doi: 10.1371/journal.pntd.0006391 (PMC5944909; doi:10.1371/journal.pntd.0006391)
Supplement: S1 File — (PDF) [file pntd.0006391.s002.pdf]

إستبان لدراسة مدى معرفة العاملين بالمهن الصحية بمرض المايستوما 2017

الاستبان القبلي

المحور الاول

المعلومات العامة:

المنطقة:...

1. **النوع:** أ.(ذكر) ب.(أنثى)
2. **العمر:**..
3. **الوظيفة:** أ.(ممرض) ب.(مساعد طبي) ت.(أخرى) حدد ....
4. **فترة العمل:**...
5. **مناطق العمل:**...

....

المحور الثاني

أسئلة المعرفة:

6. **هل تعرف مرض المايستوما(النبت- المادورا):** أ. نعم ب. لا
7. **إذا كانت الاجابة بنعم فما هو مرض المايستوما؟**

أ. مرض فطري ب. مرض بكتيري ت. مرض فيروسي ث. مرض فطري و بكتيري معاً ج. لا أعرف

8. **ما هو الميكروب المسبب لمرض المايستوما(النبت- المادورا):**

أ. التربة ب. المياه ت. لا أعرف. ث. أخرى................

9. **كيف ينتقل مرض المايستوما(النبت أو المادورا) الى جسم الانسان:**

أ. نتيجة إصابة أو جرح بالجلد حيث تدخل الميكروب من التربة.
ب. نتيجة لوخز شوكة أو أي ألة حادة تدخل معها الميكروب الى جسم الانسان
ت. نتيجة الاحتكاك بالتربة (النظافة بعد قضاء الحاجة عند الاطفال)
ث. كل ما ذكر

10. **هل مرض المايستوما معدي؟**

أ.نعم ب. لا ت. لا أعرف

11. **ما هي أجزاء جسم الانسان التي يمكن أن تتعرض للإصابة بمرض المايستوما:**

أ. الأرجل ب. الايادي ت. منطقة الظهر ث. منطقة الرأس ج. منطقة البطن
د. كل ما ذكر

12. **ما هو علاج مرض المايستوما:**

أ. علاج بلدي أو شعبي ب. علاج طبي ث. علاج طبي و شعبي معاً

13. **ما هي المخاطر التي يمكن أن يتعرض لها المريض في حالة الإهمال أو عدم المعرفة بالمرض:**

أ. زيادة حجم الاورام لدرجة قد تصعب معها حركة المريض (فقد المريض القدرة على الحركة)
ب. التعرض لالتهابات حادة
ت. وصول المرض الى العظم مما قد يؤدي لبتر العضو المصاب

14. **ما هي طرق الوقاية من مرض المايستوما:**

أ. لبس الحذاء المناسب أثناء العمل في الحواشات و الزرائب
ب. في حالة الوخز بالشوك النظافة الفورية للمنطقة و مقابلة الطبيب
ت. أخرى...

15. **ما هو موقفك تجاه مرض المايستوما ؟**

أ. إيجابي (يمكن علاجه) ب. سلبي (لايمكن علاجه)

16. **إذا كان موقفك إيجابي فما هي الاسباب:**

أ. يمكن الوقاية منه
ب. توفر العلاج المناسب
ت. أخرى...

17. **ما هو موقفك تجاه لبس الحذاء أثناء العمل في (الحواشات- زرائب البهائم) لتقليل الاصابة بمرض المايستوما:**

أ. إيجابي (ضرورة لبس الحذاء)
ب. سلبي(ليس هناك ضرورة للبس الحذاء)

18. ما هي العوامل المهمة في تحديد إشتباه مريض المايستوما؟

أ. عمل المريض

ب. البيئة المحيطة بالمريض

ت. التعرض لإصابة سابقة

ث. كل ما ذكر

19. ما هي أعراض مرض المايستوما (النبت- المادورا):

أ. ظهور أورام صغيرة تحت الجلد غير مؤلمة

ب. ظهور تقرحات على الجلد

ت. ظهور تقرحات و خروج حبيبات

ث. ظهور ورم و تقرحات و حبيبات

20. كيف يتم تشخيص مرض المايستوما؟

أ. الكشف السريري

ب. الموجات فوق الصوتية

ت. الفحص المجهري

ث. كل ما ذكر

21. كيف يتم علاج مرض المايستوما:

أ. بأستخدام الادوية فقط

ب. إجراء العمليات الجراحية

ت. إستخدام الادوية و العمليات الجراحية معاً

22. هل تقوم بإجراء عمليات لإزالة الورم : أ. نعم ب. لا

إذا كانت الاجابة بنعم ما هو نوع التخدير المستخدم؟ ....

23. متى يتم تحويل المريض لمركز أبحاث المايستوما بمستشفى سوبا الجامعي؟

أ. لإجراء الفحوصات التشخيصية للمرض

ب. في حالة عدم إستجابة المريض للعلاج

ت. في حالة تشخيص المريض بمرحلة متقدمة من المرض

ث. في حالة إحتاج المريض لإجراء عملية جراحية

ج. كا ما ذكر

إستبان لدراسة مدى معرفة العاملين بالمهن الصحية بمرض المايستوما 2017

الاستبان البعدي

المحور الاول

المعلومات العامة:

المنطقة:...

1. **النوع:** أ.(ذكر) ب.(أنثى)
2. **العمر:**...
3. **الوظيفة:** أ.(ممرض) ب.(مساعد طبي) ت.(أخرى) حدد ....
4. **فترة العمل:**...
5. **مناطق العمل:**...

....

المحور الثاني

أسئلة المعرفة:

6. **هل تعرف مرض المايستوما(النبت- المادورا):** أ. نعم ب. لا
7. **إذا كانت الاجابة بنعم فما هو مرض المايستوما؟**

أ. مرض فطري ب. مرض بكتيري ت. مرض فيروسي ث. مرض فطري و بكتيري معاً ج. لا أعرف

8. **ما هو الميكروب المسبب لمرض المايستوما(النبت- المادورا):**

أ. التربة ب. المياه ت. لا أعرف. ث. أخرى................

9. **كيف ينتقل مرض المايستوما(النبت أو المادورا) الى جسم الانسان:**

أ. نتيجة إصابة أو جرح بالجلد حيث تدخل الميكروب من التربة.

ب. نتيجة لوخز شوكة أو أي ألة حادة تدخل معها الميكروب الى جسم الانسان

ت. نتيجة الاحتكاك بالتربة (النظافة بعد قضاء الحاجة عند الاطفال)

ث. كل ما ذكر

.19 هل مرض المايستوما معدي؟

أ.نعم ب. لا ت. لا أعرف

.20 ما هي أجزاء جسم الانسان التي يمكن أن تتعرض للإصابة بمرض المايستوما:

أ. الأرجل ب. الايادي ت. منطقة الظهر ث. منطقة الرأس ج. منطقة البطن
د. كل ما ذكر

.21 ما هو علاج مرض المايستوما:

أ. علاج بلدي أو شعبي ب. علاج طبي ث. علاج طبي و شعبي معاً

.22 ما هي المخاطر التي يمكن أن يتعرض لها المريض في حالة الإهمال أو عدم المعرفة بالمرض:

أ. زيادة حجم الاورام لدرجة قد تصعب معها حركة المريض (فقد المريض القدرة على الحركة)
ب. التعرض لالتهابات حادة
ت. وصول المرض الى العظم مما قد يؤدي لبتر العضو المصاب

.23 ما هي طرق الوقاية من مرض المايستوما:

أ. لبس الحذاء المناسب أثناء العمل في الحواشات و الزرائب
ب. في حالة الوخز بالشوك النظافة الفورية للمنطقة و مقابلة الطبيب
ت. أخرى..

.24 ما هو موقفك تجاه مرض المايستوما ؟

أ. إيجابي (يمكن علاجه) ب. سلبي (لايمكن علاجه)

.25 إذا كان موقفك إيجابي فما هي الاسباب:

أ. يمكن الوقاية منه
ب. توفر العلاج المناسب
ت. أخرى..

.26 ما هو موقفك تجاه لبس الحذاء أثناء العمل في (الحواشات- زرائب البهائم) لتقليل الاصابة بمرض المايستوما:

أ. إيجابي (ضرورة لبس الحذاء)
ب. سلبي(ليس هناك ضرورة للبس الحذاء)

27. ما هي العوامل المهمة في تحديد إشتباه مريض المايستوما؟

أ. عمل المريض

ب. البيئة المحيطة بالمريض

ت. التعرض لإصابة سابقة

ث. كل ما ذكر

20. ما هي أعراض مرض المايستوما (النبت- المادورا):

أ. ظهور أورام صغيرة تحت الجلد غير مؤلمة

ب. ظهور تقرحات على الجلد

ت. ظهور تقرحات و خروج حبيبات

ث. ظهور ورم و تقرحات و حبيبات

22. كيف يتم تشخيص مرض المايستوما؟

أ. الكشف السريري

ب. الموجات فوق الصوتية

ت. الفحص المجهري

ث. كل ما ذكر

23. كيف يتم علاج مرض المايستوما:

أ. بأستخدام الادوية فقط

ب. إجراء العمليات الجراحية

ت. إستخدام الادوية و العمليات الجراحية معاً

24. هل تقوم بإجراء عمليات لإزالة الورم : أ. نعم ب. لا

إذا كانت الاجابة بنعم ما هو نوع التخدير المستخدم؟ ....

25. متى يتم تحويل المريض لمركز أبحاث المايستوما بمستشفى سوبا الجامعي؟

أ. لإجراء الفحوصات التشخيصية للمرض

ب. في حالة عدم إستجابة المريض للعلاج

ت. في حالة تشخيص المريض بمرحلة متقدمة من المرض

ث. في حالة إحتاج المريض لإجراء عملية جراحية

ج. كا ما ذكر
