# Supplementary figures and images for: A holistic approach to the mycetoma management
Source: PLoS Negl Trop Dis. 2018 May 10;12(5):e0006391. doi: 10.1371/journal.pntd.0006391 (PMC5944909; doi:10.1371/journal.pntd.0006391)

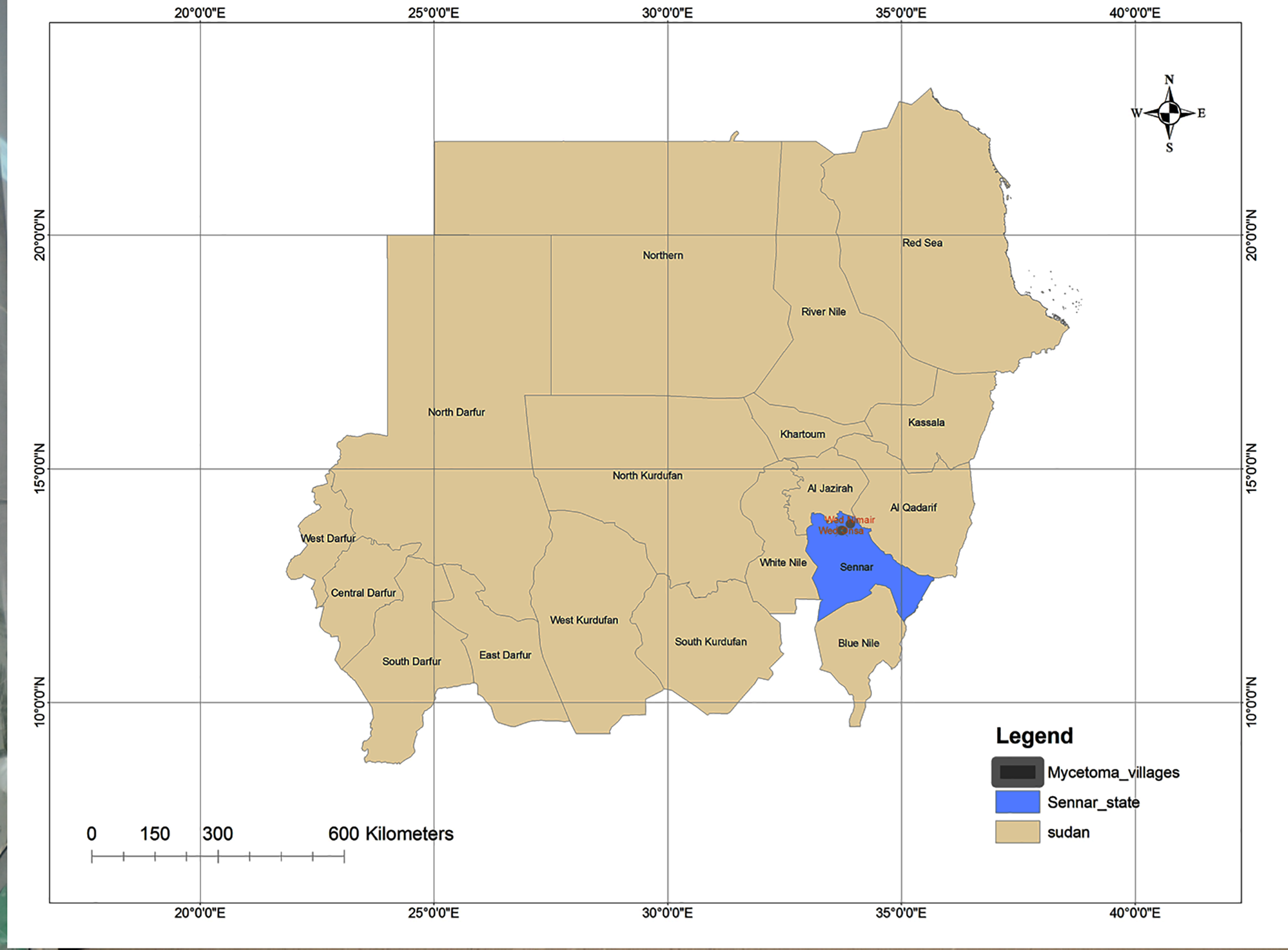

Supplement: S1 Map — (TIF) [file pntd.0006391.s003.tif]
